# Case series on the Charcot neuroarthropathy in hands after cervical central cord syndrome

**DOI:** 10.1186/s12891-022-05502-7

**Published:** 2022-06-06

**Authors:** Wenting Wang, Anni Tong, Nan Liu, Fin Biering-Soerensen, Shuai Tong

**Affiliations:** 1 Department of Rehabilitation Medicine, Beijing Haidian Hospital, Beijing, China; 2 Department of Rehabilitation Medicine, Peking University Third Hospital Haidian Division, Beijing, China; 3 Department of Rehabilitation Medicine, Peking University Third Hospital, Beijing, China; 4 Clinic for Spinal Cord Injuries, Rigshospitalet, University of Copenhagen, Copenhagen, Denmark

**Keywords:** Charcot neuroarthropathy, Spinal cord injury, Hand, Tetraplegia, Central cord syndrome, Case series

## Abstract

**Background:**

Charcot neuroarthropathy (CNA) of the upper extremity occurs most frequently in shoulders. However, CNA in the hands is uncommon and seldom be reported. The onset of CNA is usually insidious. If this process continues undetected, it can result in joint deformity, ulceration and/or superinfection, loss of function, and amputation or even death. In this article, we are going to present three cases of CNA in the hands of individuals with cervical spinal cord injury (SCI) with central cord syndrome.

**Case presentation:**

Three male individuals with cervical spinal stenosis contracted tetraplegia (American Spinal Injury Association Impairment Scale Grade D, D, and B) due to spinal cord contusion after a trauma and developed hand swelling without pain 2 to 3 months after their SCI. X-ray showed degenerative joint changes in the hands. CNA was considered due to the patient’s history of cervical SCI, loss of motor function and sensation, symptoms of painless swelling, physical examination, and X-ray findings. The self-care sub scores of Spinal Cord Independence Measure III improved slightly only during rehabilitation and follow-up due to poor hand function.

**Conclusions:**

CNA may develop after a central or peripheral neurological disorder. Nearly every joint of the body can be affected and the lower limbs are the most frequently involved. However, CNA of the hand is rare. We present three patients with CNA in the hands after cervical SCI and review the features and early differential diagnosis of CNA. Currently there is no specific treatment available. Therefore, early identification of CNA and adequate protection to the affected joints seem important.

## Background

Charcot neuroarthropathy (CNA) was first described by Jean Marie Charcot in 1868, and refers to progressive degeneration of a joint. It featured with bony destruction, bone resorption due to loss of sensation because of central nervous system or peripheral nerve damage. The onset of CNA is usually insidious. If this process continues undetected, it can result in joint deformity, ulceration and/or superinfection, loss of function, and amputation or even death. Early identification of joint changes is the best way to decrease risk of complications. CNA of the upper extremity occurs most frequently in shoulders [1]. However, CNA in the hands is uncommon. In this study, we present three cases of CNA in the hands of individuals with cervical spinal cord injury (SCI), and discus the differential diagnosis in order to enhance early detection and facilitate appropriate protective management.

## Case presentation

### Case 1

A 74-year-old male with C4 American Spinal Injury Association Impairment Scale (AIS) grade D tetraplegia was admitted to the SCI unit 1 week after laminoplasty for decompression. He had a history of cervical stenosis and sustained a cervical SCI due to spinal cord contusion after a fall during walking. At admission, neurological examination according to the International Standards for Neurological Classification of Spinal Cord Injury (ISNCSCI) revealed his injury pattern was in accordance with the diagnostic criteria of central cord syndrome (CCS) [2], i.e. an average of 10 muscle points less in the upper extremities motor scores (UEMS) compared to the lower extremities motor scores (LEMS), as the UEMS was 12 and the corresponding for the LEMS was 37. Both sensation for pin prick (PP) and light touch (LT) of C6, C7 and C8 dermatomes were decreased. The Spinal Cord Independence Measure (SCIM III) total scores are given in Table 1.

**Table 1:** The Spinal Cord Independence Measure (SCIM III) score of three cases

	Admission	Discharge	Follow-up
**CASE 1**			
**Time since SCI**	1 week	6 months	24 months
Self-care sub-score	0	1	1
Respiration & sphincter management	15	18	28
Mobility sub-score	0	18	35
total SCIM score	15	36	64
**CASE 2**			
**Time since SCI**	1 week	6 months	15 months
Self-care sub-score	3	6	10
Respiration & sphincter management	26	31	31
Mobility sub-score	0	16	10^a^
total SCIM score	29	53	51
**CASE 3**			
**Time since SCI**	1 week	6 months	10 months
Self-care sub-score	0	4	5
Respiration & sphincter management	18	29	32
Mobility sub-score	0	8	13
total SCIM score	18	41	50

Spinal Cord Independence Measure (SCIM III) score of three patients with spinal cord injury (SCI) at admission, discharge and follow-up

^a^see text

The resting blood pressure (BP) monitoring at admission was 113/68 mmHg, much lower than that before surgery. The patient had a history of hypertension for more than 30 years and the average BP was 150/90 mmHg before SCI. With the lowest BP of 81/50 mmHg during tilt bed test, the patient had no symptoms of orthostatic hypotension (OH) such as dizziness and faint. Two months after SCI, the metacarpophalangeal (MCP) and proximal and distal interphalangeal (PIP and DIP) joints of both hands gradually became swollen and warm, the patient complained mild discomfort without pain or sweating. Ice therapy was given with poor effect.

**Fig. 1:**
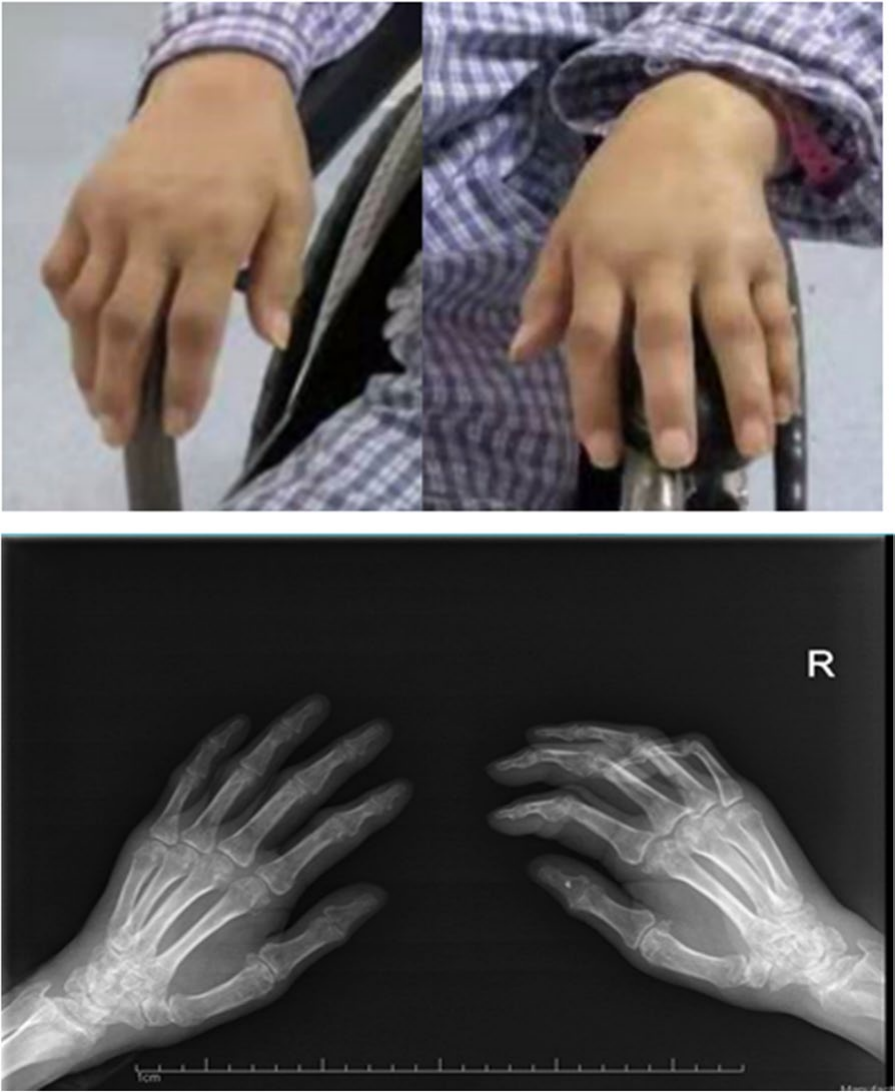
Charcot neuroarthropathy in hands after cervical central cord syndrome: six months after spinal cord injury, painless swellings (above) and degenerative changes of both proximal and distal interphalangeal joints at X-ray of the hands (below)

After 5 months rehabilitation, walking function of the patient improved significantly, and the SCIM III mobility sub-score increased from 0 to 18. However, recovery of self-care sub-score was subtle (from 0 to 1). Although bilateral shoulder and elbow range of motion (ROM) was normal, the wrist flexion and extension was poor, together with decreased passive ROM (PROM) of the MCP, PIP and DIP joints. The patient didn’t complain any pain, but had swelling, deformity of the MCP, PIP and DIP joints, dry rough skin and unchanged decreased PP and LT sensation in both hands. X-ray showed that there were degenerative changes at the edge of the MCP, PIP and DIP joints. The joint surfaces were not smooth, the joint spaces were narrow, and there were high-density spot-like shadows at the distal phalanx of the right thumb (Fig. 1). The patient claimed not to have had any joint problems before his SCI.

At the follow-up 2 years post injury, swelling in both hands had diminished, but the patient still could not perform e.g. eating, bathing, grooming, and dressing. Although muscle group function around shoulders was normal, and muscle strength in the upper extremities recovered in the majority of the key-muscle functions as following: normal in elbow flexor (5/5) and extensor (5/5), wrist extensor (3/3), finger flexors (2/2) and adductor (2/2), it still contributes to a SCIM self-care sub-score as low as 1 (Table 1). The sensory examinations according the ISNCSCI were the same as at admission. There were active movements over the MCP joints, and the PIP joints could actively flex to 90° while the DIP joints could flex 40° only.

For patient no. 1, the most distinctive feature was severe joint swelling and destruction in DIP and PIP joints of both hands without pain, and there was weakness in both arms and hands. For these reasons, he couldn’t accomplish balance or transfer training, and could not ambulate using a walker in early phase of rehabilitation. He felt sad although there was no pain or discomfort in the hands. He still couldn’t administrate bowel or bladder routines due to poor hand function, and intermittent catheterization had to performed by a caregiver.

### Case 2

A 71-year-old male with C3 AIS D tetraplegia with cervical stenosis suffered spinal cord contusion due to a traffic accident. Laminoplasty for decompression was conducted 6 hours after SCI. He was admitted to the SCI rehabilitation unit 1 week after injury, neurological examination according to the ISNCSCI revealed the total motor score was 54, UEMS and LEMS were 28 and 26 respectively. Hyperesthesia was found from C6 to C8 on the right side, while on the left side hypoesthesia was found at C6 and absent sensation for PP and LT at C7 and C8 dermatomes.

The patient complained of pain in his right shoulder, upper limb and hand, which was characterized by tingling and burning pain. It was diagnosed as neuropathic pain according to Douleur Neuropathique 4 questionnaire, and treated with pregabalin. Meanwhile, hyponatremia (the lowest serum sodium level was 125 mmol/L) and OH appeared. Oral salt supplement was given with 6 g/day of sodium chloride and the serum sodium returned to normal 1 month after admission. When “standing” at 40° during tilting table excises, the patient complained dizziness, fainting and coat-hanger pain in the right shoulder with the lowest BP of 88/54 mmHg. Three months after SCI, the patient could keep upright posture for 30 mins without dizziness or fainting. At this time the MCP, PIP and DIP joints of the right hand became warm and swollen, but the patient complained little discomfort only and sweating without pain. The manual lymphatic drainage technology (MLDT) was conducted which diminished the swelling for few hours only.

Six months after SCI, the MCP, PIP, and DIP joints of the right hand were still swollen while there was no swelling of the left hand. The neurological examination according to the ISNCSCI revealed the total motor score was 67, UEMS and LEMS were 28 and 39 respectively meeting the criteria of CCS. The sensation of C6–8 dermatomes was unchanged from time of admission. X-ray showed degenerative changes in right hand with hyper osteogenesis and sclerosis at the edge of the MCP, PIP, and DIP joints, part of the joint spaces were slightly narrow, and high-density spot-like shadows near the joint spaces were found. There were no degenerative changes on the X-rays of shoulders, wrists and the left hand. The patient could not perform activities such as eating, grooming, dressing, and bathing independently. Walking function improved significantly which was shown on the SCIM III mobility sub-score increased from 0 to 16, but the self-care sub-score improved only slightly from 3 to 6 (Table 1).

At the follow-up 15 months post injury, swelling of the right hand had resolved, but the patient still could not perform ADLs such as feeding and grooming with the right hand although he was right-handed before injury, but he could eat and groom independently using the left hand, indicated by SCIM III self-care sub-score increased from 6 to 10. The sensation did not change. However, the mobility sub-score decreased from 16 to 10 due to lack of supervision at home and fear of falling, hence the total SCIM III score decreased from 53 to 51 (Table 1).

### Case 3

A 51-year-old male with C4 AIS B tetraplegia who also had a history of cervical stenosis sustained spinal cord contusion after a fall. The patient had ankylosing spondylitis (AS) and diabetes for 10 years. At admission, neurological examination, according to the ISNCSCI, revealed hypoesthesia from C6 to C8 on the left and both PP and LT were normal on the right hand. The strengths of both elbow flexors were grade 1 while other key-muscle functions in the upper extremity were 0.

The patient complained painless joint swelling, movement limitation of both hands 2 months after injury. Physical examination revealed that the ROM of MCP, PIP, and DIP joints of both hands were limited because of swelling, while the patient did not complain any pain neither in process of PROM exam nor when applying pressure to the joints. Wrist ROM was normal. X-ray showed degenerative changes in PIP and DIP joints on both hands but no changes in the shoulders, elbows, wrists or the feet. The results of blood analysis including the anti-streptococcal hemolysin-O, C-reactive protein (CRP), serum erythrocyte sedimentation rate, uric acid (UA), anti-nuclear antibody (ANA), extractable nuclear antigens (ENA), free T3, free T4, thyroid stimulating hormone, blood glucose and the Hemoglobin A1c were normal, while the rheumatoid factors (RF) value was increased to 44 U/mL (normal range: 0–25 U/mL). The human leukocyte antigen was positive. It was concluded the swelling of the hands was not related to activity of his AS.

Six months after SCI, UEMS had improved from 2 to 26 but the swelling persisted while the sensation did not change. The patient could not eat independently because of weakness of grip function, but he could take on clothes without buttons, zippers or lace-up when partially assisted. The SCIM III self-care sub-score improved from 0 to 4 (Table 1). The LEMS was improved from 0 to 38 and he could stand independently with a walker, although the body was bent forward because of AS. He could not step forward with walker because of weak grasp strength of both hands. The SCIM III mobility sub-score improved from 0 to 8 (Table 1).

At follow-up 10 months post injury, swelling persisted, and the SCIM III self-care sub-score had increased slightly to 5. The patient could operate the wheelchair forward and backward with his legs indoor, and could walk up and down at least 3 stairs with the help from one person. The SCIM III mobility sub-score increased to 13 and the total SCIM score increased to 50 (Table 1).

The symptoms and laboratory tests of the three patients were recorded in Table 2.

**Table 2:** The symptoms and laboratory tests of the three patients

	Case 1	Case 2	Case 3
**Leukocytosis**	no	no	no
**Erythrocyte sedimentation rate (ESR)**	normal	normal	normal
**C-reactive protein (CRP)**	normal	normal	normal
**Hyperglycemia**	no	no	Type 2 diabetes was diagnosed 10 years ago, but blood glucose and Hemoglobin A1c monitoring were normal after admission.
**Clinical inflammation**	no	no	no
**Other Arthropathy**	no	no	History of ankylosing spondylitis
**History of traumatic SCI**	yes	yes	yes
**Course of disease**	2 months	3 months	2 months
**Bony deformities**	degenerative changes at the edge of the MCP, PIP and DIP joints	degenerative changes in right hand with hyper osteogenesis and sclerosis at the edge of the MCP, PIP, and DIP joints	degenerative changes in PIP and DIP joints on both hands but no changes in the shoulders, elbows, wrists or the feet
**Signs of neurological or vascular deficiency**	yes	yes	yes
**Neurological findings**			
**Numbness**	no	no	no
**Dysaesthesia**	yes	yes	yes
**Changes in the texture of the skin**	yes	yes	yes
**Cutaneous changes**			
**Sweating**	yes	yes	yes
**Calluses**	yes	yes	yes
**Temperature**	worm	worm	worm
**Color**	dull	dull	dull
**Tendon reflex**	+++	+++	+++
**Vibration sense**	hypesthesia	normal	hypesthesia
**Cutaneous sensitivity**	not test	not test	not test

## Discussion and conclusion

In this study, we present three patients with traumatic tetraplegia with hand CNA, which to our knowledge has never been reported before. CNA is commonly seen in medical and neurological diseases such as diabetic neuropathy [3], tertiary syphilis, cerebral palsy, syringomyelia, SCI, etc. It is characterized by the degeneration and painless swelling of the joints. The joints most frequently affected are the weight-bearing joints, predominantly the mid-foot and then ankle [4], knee, hip and rarely the spine [5] and upper extremity, especially seldom in hands [6]. The involved joints are partly dependent on the underlying neurological condition such as foot and ankle in diabetes [7], knees in tabes dorsalis [8], upper extremity joints in syringomyelia [9] and hand in syringomyelia [10]. However, the cases reported in individuals with SCI were commonly in the spine know as Charcot spine arthropathy [11, 12]. This article may deepen the understanding of CNA, and remind us that we should not only focus on those general cognized weight-bearing joints, but also should care about joints in potential dangerous, such as cervical SCI patients’ hands, which may become vulnerable and have to replace part of the role of weight-bearing. Since there is no effect way in CNA’s treatment, early diagnose and then give adequate protection seem extremely important.

In CNA the joint lack the protective sensation when exposed to abnormal stress, leading to destruction of joints and surrounding bony structures. Consequently, the subchondral bone of the involved joint disintegrates, which may lead to joint collapse and deformity. All three patients we reported had different degrees of sensory impairment, and did not recover or improve during the rehabilitation processes. Even though the skin sensation of patient 2 was normal in one hand, it may not imply that the deeper sensation is normal, supported by no pain was felt when pressure was applied to the respective joints. When using the hands at early stage performing transfer training or weight shifting, the patients could not react protective to abnormal loads on the hands, which in part may be the reason for CNA did develop.

CNA may be staged according to the modified Eichenholtz Classification: Stage 0, there is joint edema, warmth and erythema, radiographs are negative; Stage 1, there is joint edema, radiographs shows osseous fragmentation with joint subluxation or dislocation; Stage 2, local edema, warmth and erythema decreased, radiographs shows coalescence of fragments and absorption of fine bone debris; Stage 3, there is no local edema, radiographs shows consolidation and remodeling of fracture fragments [13].

X-ray in middle stage can show the degenerative change of the involved joint, then obliteration of joint space, fragmentation of both articular surfaces of the joint leading to subluxation or dislocation, scattered “chunks” of bone in fibrous tissue, joint distention by fluid, surrounding soft tissue edema and heterotopic ossification can develop. When plain radiographs do not show any initial signs of osseous fragmentation or dislocation in spite of clinical suspicion, other imaging methods, including magnetic resonance imaging (MRI), computed tomography, and bone scintigraphy, may support the diagnosis [14]. E.g., at stage 0, soft tissue edema and subchondral bone marrow edema may be found with MRI.

Hands of the three patients all showed different degrees of swelling and limited joint activity. X-ray indicated degenerative changes of the joints, which was consistent with the diagnosis of stage 1 or 2 of the modified Eichenholtz Classification. In the three patients, painless swelling of the hands occurred 2–3 months after injury, which may indicate the beginning of CNA. We believe that there is no strict time limit between the three stages. Maybe there was degenerative changes of the joint on the X-ray when the patient developed swelling, but we did not conduct X-ray examination at the initial stage. The swelling may diminish or disappear from 15 to 24 months after SCI. The final diagnosis should be a combination of symptoms and X-ray findings.

In patients with CCS from traumatic cervical SCI, the upper extremities are more severely affected than the lower extremities, which may contribute to upper limbs being more susceptible developing CNA. The three patients reported eventually all met the diagnostic criteria of CCS. This suggests that, for patients with tetraplegia and may in particular with CCS, once joint swelling of the hands occurs, CNA should be thought about and other potential causes including Complex reginal pain syndrome (CRPS), osteoarthritis or rheumatoid arthritis (RA) need to be ruled out as CNA is a diagnosis of exclusion.

Autonomic nervous system dysfunction after SCI may also play a role in the development of CNA. In cervical and high thoracic injuries, loss of supraspinal regulatory control of the sympathetic nervous system may result in reduced overall sympathetic activity below the level of injury and cause conditions such as hypotension, bradycardia, and a blunted cardiovascular response to exercise [15]. Two of the three patients presented experienced OH and hypohydrosis below the level of injury, which is a manifestation of autonomic dysfunction mainly at the early stage after injury, although patient no. 1 had no symptoms. In addition, for patient no. 2, CNA of the right hand was more severe than for the left hand and the OH related coat-hanger pain also emerged at the right side. Therefore, it is suggested that patients with cervical SCI combined with autonomic dysfunction may be more likely to develop CNA.

CRPS is a kind of neuropathic pain disorder that develop as the exaggerated response to a traumatic lesion that in general affect the extremities, or is a consequence of an event such as a stroke. CRPS of the upper arm after stroke is still frequently known as shoulder-hand syndrome [16]. It’s relevant to the sympathetic nervous system and has two forms, that are CRPS 1 and CRPS 2 respectively. It may be confused with hand CNA as both have hand swelling and stiffness. However, it can be distinguished from X-ray as CRPS will not show degenerative changes.

There is also evidence for a relationship between CNA and RA [17]. Patient no. 3 had AS which is another type of autoimmune disease. Thus, a possible differential diagnosis between AS and CNA. The usual clinical manifestation of CNA is a painless joint swelling, or mild discomfort, if any, and the symptoms are disproportionately mild in relation to the degree of destructive joint changes seen radiographically [18]. Some patient complaint loss of function. Physical exam reveals swollen, warm, and erythematous joints, similar to infection. But the results of blood analysis such as the RF, CRP, UA, ANA, ENA, etc. are normal. The joint of CNA can be mechanically unstable because of impaired sensation and limited active range of motion.

According to the literature, the key to successful management of CNA depends on a thorough history and examination along with specific laboratory testing and imaging to rule out other relevant diseases [1]. There is yet no specific pharmacological treatment for CNA. Some scholars suggest early application of bisphosphonates to improve bone mineral density in diabetic patients [19]. Once diagnosed, rest, elevation, protected immobilization with a sling, and restriction of activity should be conducted. NSAIDs may be a choice for acute inflammatory stage. For physical therapists, CNA should allow flexion-extension in neutral position. Charcot joint is a contraindication to total joint replacement due to poor bone stock, prosthetic loosening, instability, and soft-tissue compromise. The goal in Charcot foot treatment is to obtain and maintain the correction of a severe deformity and/or prevent its development [20]. But the treatment for Charcot’s hand has not been reported. The MLDT could be performed to release swelling. It is recommended that therapists should be aware that transfers and other training that may put physical stress to the hand joints should be avoided. In addition.

Three cases of hands CNA have been reported in this article. When the joints of hand swelling occur in the early stage during rehabilitation training for patients with tetraplegia, it is recommended to perform X-ray examination as soon as possible to determine whether CNA is existing. The limitation of this report is that we did not track the X-ray changes in the subsequent follow-up, and it is not clear whether the X-ray changes in the joints grew to the next stage(s) according to the diagnostic criteria mentioned above. Therefore, further prospective researches are warranted to determine the progress and prognosis.

The reported individuals with tetraplegia presented painless swelling of the metacarpophalangeal and interphalangeal joints in the hands without obvious causes, accompanied by limited movement, inability to grasp and take care of themselves in daily life. X-ray of the hand showed osteophytes, hypertrophic, erosive and destructive changes on the MCP, PIP, and DIP joints. CNA is irreversible and the joint lesions may aggravate. There is currently no specific treatment available. So, early diagnose and then give adequate protection seem extremely important. If there are early joint changes, it should be protected in the training process to avoid excessive joint loading, which will lead to the aggravation of degenerative changes and further affect the recovery of upper limb and hand function in the later period. When using the hands at early stage performing transfer training or weight shifting, the patients could not react protective to abnormal loads on the hands, which in part may be the reason for CNA. Besides, it is important to educate patients and their family members how to protect the affected joints as these patients are not able to feel the pain properly.

In summary, early detection and diagnosis of CNA are important for patients with CCS after cervical SCI. Once painless joint swelling occurs, excluding acute arthritis and other inflammatory reactions, CNA should be highly suspected. Furthermore, affected joints should be protected in the training process to avoid excessive joint loading, and it is important to educate patients and their family members as well. Unsupervised attempts to improve range of motion in a SCI patient need careful consideration.

## Data Availability

Not applicable.

## Abbreviations

CNA: Charcot neuroarthropathy
SCI: Spinal cord injury
AIS: American Spinal Injury Association Impairment Scale
ISNCSCI: International Standards for Neurological Classification of Spinal Cord Injury
CCS: Central cord syndrome
UEMS: Upper extremities motor scores
LEMS: Lower extremities motor scores
PP: Pin prick
LT: Light touch
SCIM III: Spinal Cord Independence Measure version III
BP: Blood pressure
OH: Orthostatic hypotension
MCP: Metacarpophalangeal
PIP: Proximal interphalangeal
DIP: Distal interphalangeal
ROM: Range of motion
PROM: Passive ROM
MLDT: Manual lymphatic drainage technology
AS: Ankylosing spondylitis
CRP: C-reactive protein
UA: Uric acid
ANA: Anti-nuclear antibody
ENA: Extractable nuclear antigens
RF: Rheumatoid factors
CRPS: Complex reginal pain syndrome
RA: Rheumatoid arthritis
